# Supplementary material for: Beyond flickering: introducing code-modulated motion visual evoked potentials for brain-computer interfacing
Source: Front Neuroergon. 2026 Aug 20;7:1884144. doi: 10.3389/fnrgo.2026.1884144 (PMC13538422; doi:10.3389/fnrgo.2026.1884144)
Supplement: Supplementary file 1 [file Data_Sheet_1.pdf]

Supplementary Material

1 SUPPLEMENTARY TABLES AND FIGURES

1.1 Figures

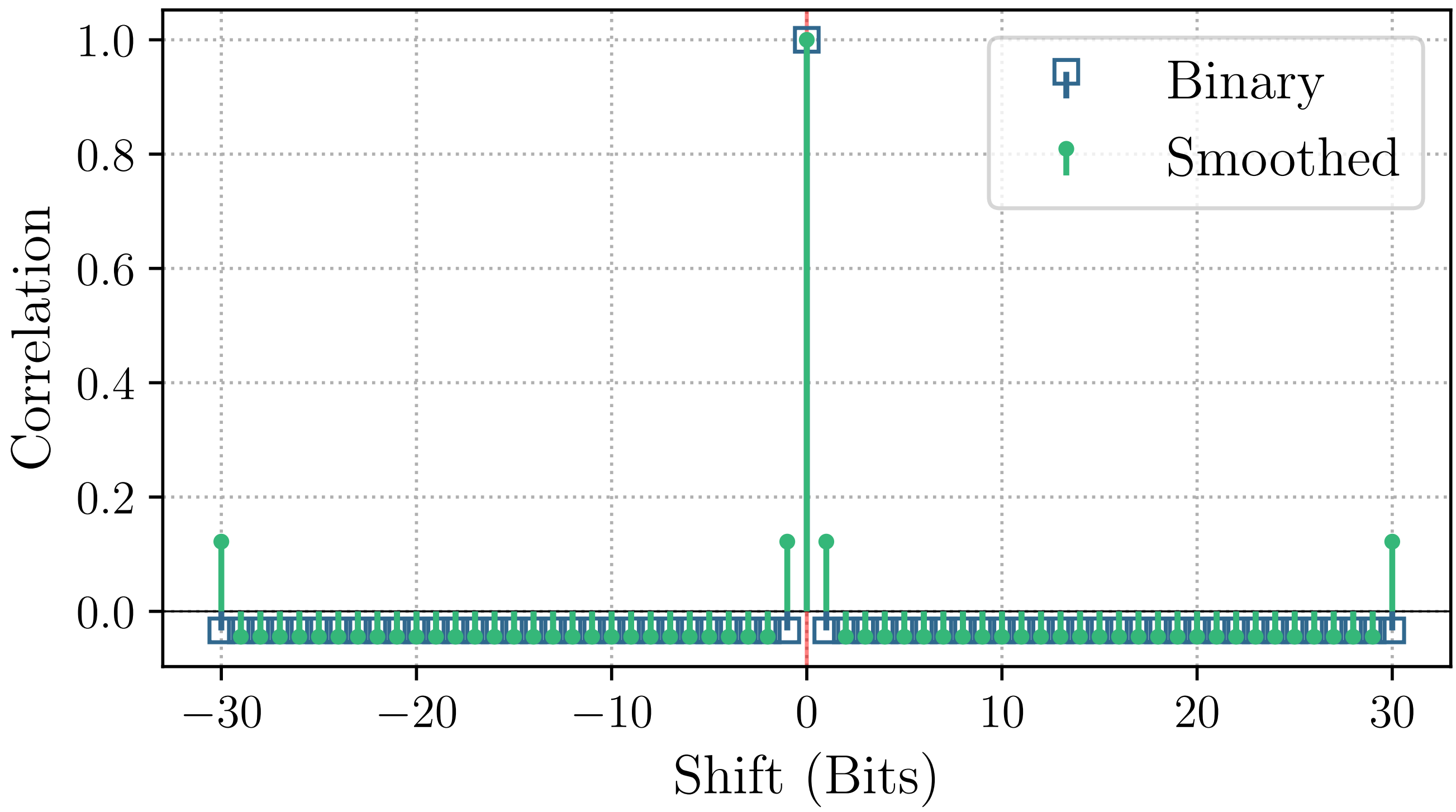

Figure S1. Periodic autocorrelation of the m-sequences. For both the original 31-bit binary m-sequence and the smoothed 31-bit m-sequence.

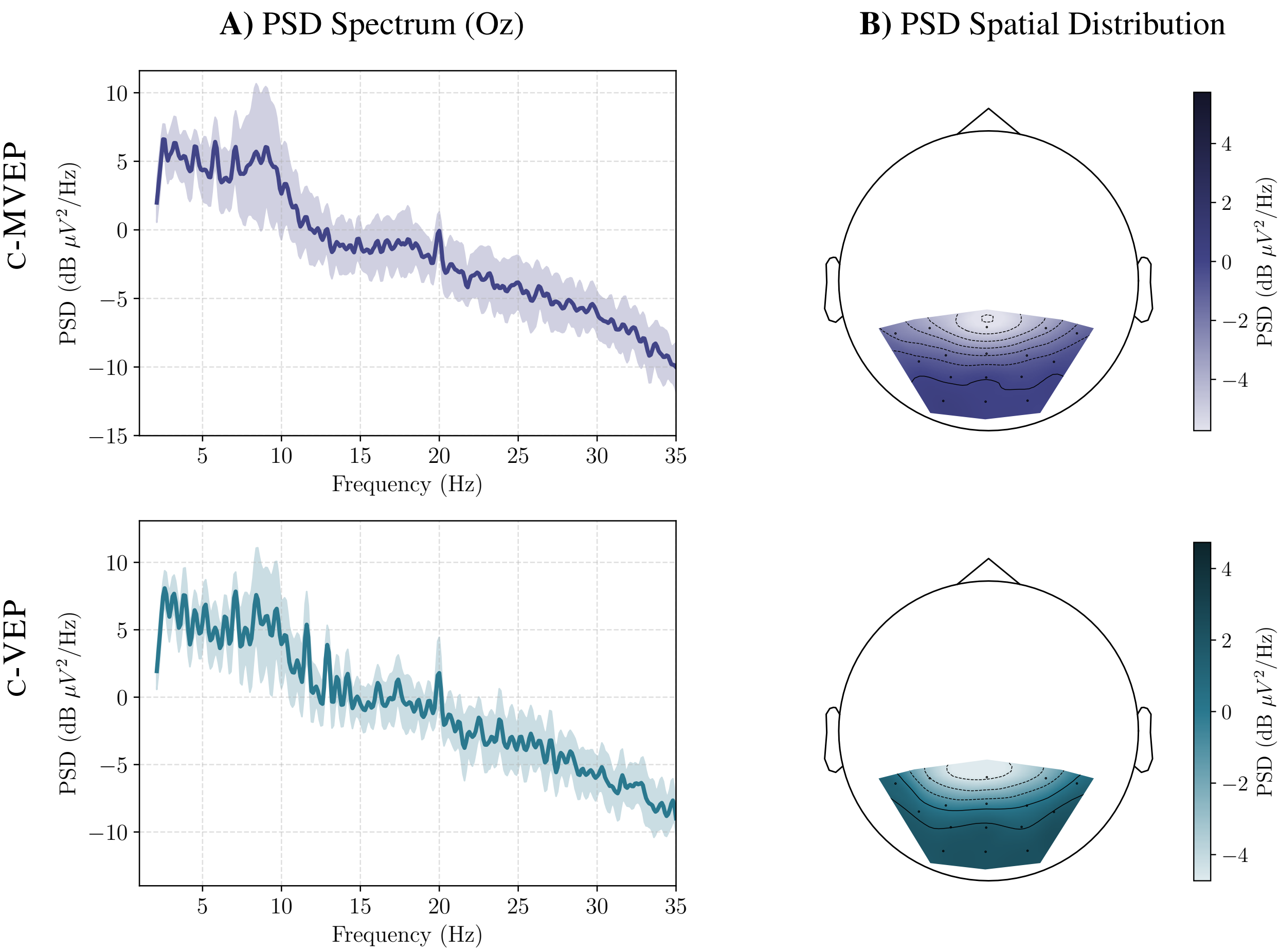

Figure S2. Signal characteristics for c-MVEP and c-VEP. The thick lines show the mean response, the shaded area the standard error. Columns show (A) the PSD spectrum at channel Oz. Column (B) shows the spatial distribution of the 20 Hz power.

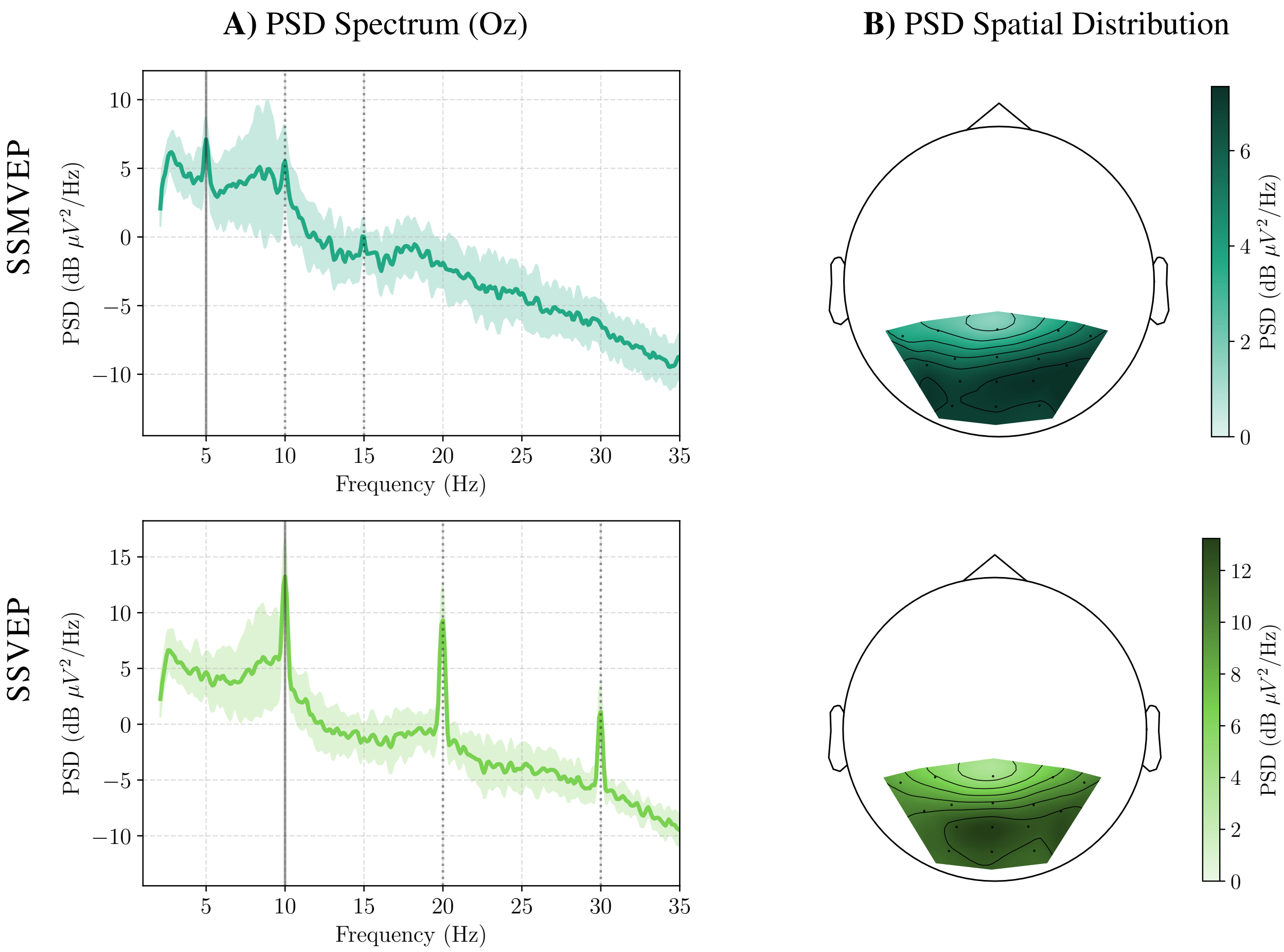

Figure S3. Signal characteristics for SSMVEP and SSVEP. The thick lines show the mean response, the shaded area the standard error. Columns show (A) the PSD spectrum at channel Oz. Column (B) shows the spatial distribution of the 5 Hz (SSMVEP) and 10 Hz (SSVEP) power.

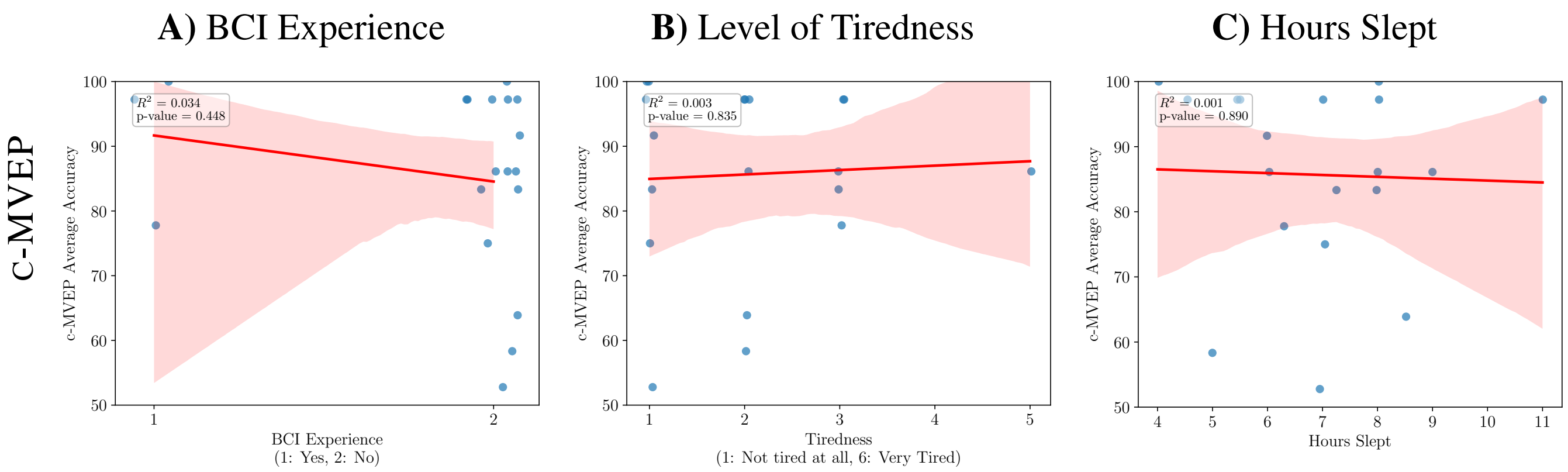

Figure S4. Predictors against Average Decoding Performance. Predictors from the pre-questionnaire plotted against the average decoding performance for c-MVEP. All jittered scatter plots were fitted with a linear regression model to visualize the relationship between the predictors and the c-MVEP average decoding performance. The predictors included were A) BCI Experience (1: Yes, 2: No), B) Level of Tiredness (1: Not tired at all, 6: Very Tired), and C) Hours Slept.

2 SUBJECTIVE QUESTIONNAIRES

1. Offline Questionnaire

For Questions 1–6, participants responded using a 6-point Likert scale.

1. Rate your eye discomfort for this stimulus.
   Scale: 1 (Very uncomfortable) – 6 (Not uncomfortable at all)
2. How easy was it to concentrate on the target?
   Scale: 1 (Very hard) – 6 (Very easy)
3. Was the flickering/moving disturbing for you?
   Scale: 1 (Very disturbing) – 6 (Not disturbing at all)
4. How often did you perceive a loss of focus during the stimulation?
   Scale: 1 (No loss at all) – 6 (Very often lost focus)
5. Would you like a BCI speller with this stimulation?
   Scale: 1 (Do not agree at all) – 6 (Very much agree)
6. Rate your overall impression of the stimulus.
   Scale: 1 (Poor) – 6 (Excellent)

2. Online Questionnaire

For Questions 1–7, participants responded using a 6-point Likert scale.

1. How tired do you feel right now?
   Scale: 1 (Not tired at all) – 6 (Very tired)
2. Rate your eye discomfort for this stimulus.
   Scale: 1 (Very uncomfortable) – 6 (Not uncomfortable at all)
3. How easy was it to concentrate on the target?
   Scale: 1 (Very hard) – 6 (Very easy)
4. Was the flickering/moving disturbing for you?
   Scale: 1 (Very disturbing) – 6 (Not disturbing at all)
5. How often did you perceive a loss of focus during the stimulation?
   Scale: 1 (No loss at all) – 6 (Very often lost focus)
6. Would you like a BCI speller with this stimulation?
   Scale: 1 (Do not agree at all) – 6 (Very much agree)
7. Rate your overall impression of the stimulus.
   Scale: 1 (Poor) – 6 (Excellent)

3. Online Post-Questionnaire

Participants selected one of the binary choices provided.

1. Which stimulation did you like the best?
   Options: 1 (Zooming), 2 (Flickering)
2. Which stimulation caused the most eyestrain?
   Options: 1 (Zooming), 2 (Flickering)

3. Which stimulation was easiest to focus on?
   Options: 1 (Zooming), 2 (Flickering)
4. Did you notice a difference between the two flashing sessions?
   Options: 1 (Yes), 2 (No)
5. Did you notice a difference between the two zooming sessions?
   Options: 1 (Yes), 2 (No)
